# Using Genetic Prediction from Known Complex Disease Loci to Guide the Design of Next-Generation Sequencing Experiments

**DOI:** 10.1371/journal.pone.0076328

**Published:** 2013-10-18

**Authors:** Luke Jostins, Adam P. Levine, Jeffrey C. Barrett

**Affiliations:** 1 Medical Genomics, Wellcome Trust Sanger Institute, Wellcome Trust Genome Campus, Hinxton, Cambridge, United Kingdom; 2 Wellcome Trust Centre for Human Genetics, Univeristy of Oxford, Oxford, United Kingdom; 3 Division of Medicine, University College London, London, United Kingdom; IFOM, Fondazione Istituto FIRC di Oncologia Molecolare, Italy

## Abstract

A central focus of complex disease genetics after genome-wide association studies (GWAS) is to identify low frequency and rare risk variants, which may account for an important fraction of disease heritability unexplained by GWAS. A profusion of studies using next-generation sequencing are seeking such risk alleles. We describe how already-known complex trait loci (largely from GWAS) can be used to guide the design of these new studies by selecting cases, controls, or families who are most likely to harbor undiscovered risk alleles. We show that genetic risk prediction can select unrelated cases from large cohorts who are enriched for unknown risk factors, or multiply-affected families that are more likely to harbor high-penetrance risk alleles. We derive the frequency of an undiscovered risk allele in selected cases and controls, and show how this relates to the variance explained by the risk score, the disease prevalence and the population frequency of the risk allele. We also describe a new method for informing the design of sequencing studies using genetic risk prediction in large partially-genotyped families using an extension of the Inside-Outside algorithm for inference on trees. We explore several study design scenarios using both simulated and real data, and show that in many cases genetic risk prediction can provide significant increases in power to detect low-frequency and rare risk alleles. The same approach can also be used to aid discovery of non-genetic risk factors, suggesting possible future utility of genetic risk prediction in conventional epidemiology. Software implementing the methods in this paper is available in the R package Mangrove.

## Introduction

Risk for many complex diseases [1], [2] can be partially predicted by a variety of proven risk factors, both genetic and environmental. While the clinical utility of these predictors is widely debated [3], the potential to use them in the design of future research has been less well studied. Specifically, can known predictors of disease susceptibility aid the discovery of new risk factors by targeting individuals for whom the presence or absence of disease is most surprising?

This question is particularly timely in the field of complex disease genetics. While it is widely known that genome-wide association studies (GWAS) have explained only a minority of variance in most complex diseases [4], the loci discovered are still able to predict many diseases as well as (or better than) established non-genetic risk factors [5]. Since GWAS were designed to detect common risk variants [6], a natural next step is to undertake studies that are able to detect low-frequency and rare risk variants using next-generation sequencing [7], [8]. As sequencing is still relatively expensive, there is much interest in choosing the optimal samples to sequence based on other information: previous studies have looked at selecting samples based on extreme phenotypes, family history, linkage information and environmental risk [9], [10]. A currently unexplored possibility, however, is to use the modest but real predictive capacity allowed by GWAS in designing these studies. Therefore, in this paper we investigate the potential power gained by using genetic risk factors established via GWAS in next-generation sequencing experiments.

A natural hypothesis is that the residual variation in phenotype, after known risk factors have been accounted for, is explained by as-yet-undiscovered factors that may include low-frequency risk alleles. Therefore, we propose the following straightforward approach: identify individuals whose observed phenotype is not well explained by their genotype. This could be applied to single affected individuals or families with a high burden of disease despite having a low genetic risk, or to those who fail to develop the disease despite having a high risk. In either case, these individuals are more likely to harbor as-yet unidentified risk factors (both genetic and environmental). For instance, it has been shown that individuals with very high LDL cholesterol with known familial hypercholesterolaemia mutations have a much lower load of common cholesterol increasing alleles than individuals with high LDL but no known monogenic cause [11].

Previous studies have implicitely used this approach to increase power by excluding cases who carry either Mendelian forms of the disease (such as MODY cases in diabetes [12]) or high penetrance risk factors (such as BRCA1/2 in breast cancer [13]). A closely related (and complementary) method is to select cases and controls on the basis of their environmental risk, which has been shown to increase power to detect low-frequency risk variants [14]. The approach we describe here can also be viewed as an extension of an extremes of quantitative traits approach, where we seek individuals with very large differences between their observed and genetically predicted values for the trait in question.

When an individual is genotyped at known GWAS loci, we use standard linear or logistic models to predict continuous or binary phenotypes [15]. We show in our first scenario below how these predictors can be used to prioritise a subset of individuals for sequencing from a large collection, with the aim of discovering low-frequency risk variants circulating in the population. For the discovery of truly rare but more penetrant variants we consider large multiply-affected pedigrees in scenario 2. We present a new method that extends the risk prediction approach to families without complete genotype data using a modification of the inside-outside algorithm for inference on trees. We perform simulations to investigate how well these methods perform in each scenario.

## Results

### The impact of prioritisation on low-frequency risk variants

Risk prediction algorithms use sets of known risk factors to predict the value of an individual's phenotype 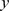 (either 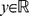 for continuous phenotypes, or 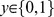 for binary phenotypes). We will refer to this predicted phenotype as 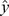, with 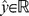 for continuous phenotype predictions and 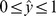 for binary phenotype predictions. While many methods for producing predicted phenotypes exist, throughout this paper we use a standard linear or logistic model to predict continuous and binary traits respectively (see Methods below).

To prioritise individuals for study, we select the individuals who have the largest difference between their actual and predicted observations 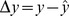, and compare those who have large positive differences to those who have large negative differences. For continuous traits this is equivalent to sampling the extremes of the distribution after factoring out the variance explained by known genetics. As with standard extreme trait analyses [16] the distribution of 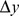 after selection follows a truncated normal distribution, and for maximum power a corrected linear analysis of 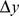 against genotypes *g* can be performed [17]. However, for simplicity we will instead consider a case-control analysis of the two extremes in this paper.

For binary disease traits, all affected individuals have 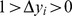, and unaffected individuals have 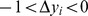, but affected individuals with low disease probability will approach 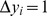, and unaffected individuals with high disease probability will have a value close to 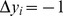 (Figure S1 in File S1 shows this distribution for diseases with different heritability and prevalence). Thus in both the quantitative and binary scenarios comparing the extremes of 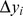 serves to identify a set of “super cases” and “super controls” who are maximally separated on an axis of unexplained risk.

**Figure 1:**
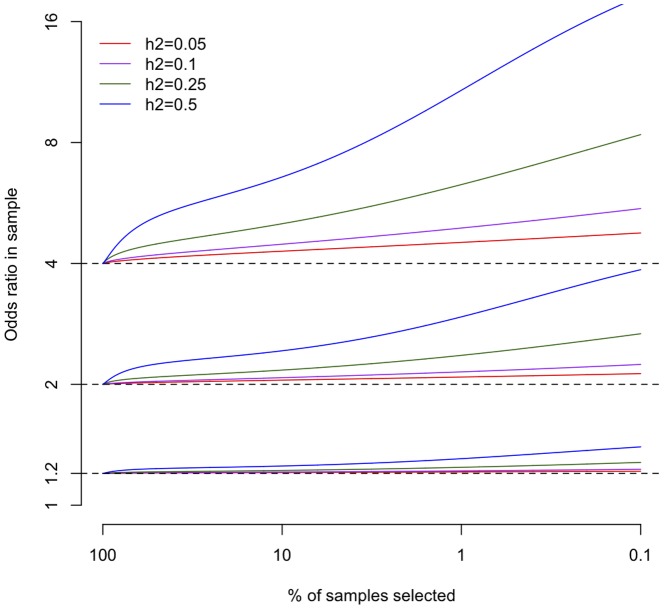
The increase in apparent odds ratio after selecting samples with 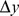 in the top or bottom 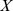
%. Coloured lines represent different amounts of variance explained by the risk score 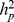. The odds ratios in unselected individuals were 1.2, 2 and 4, the disease was assumed to have prevalence 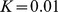, and the allele frequency was assumed to be 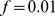.

In both cases we can calculate the allele frequency in these selected cases and controls as a function of the odds ratio and allele frequency in unselected individuals, prevalence of the disease, variance explained by the risk score and the thresholds of the risk score chosen. Details of these calculations are given in Appendix A in File S1, and the apparent odds ratio in the selected samples are given in Figure 1, and in Figure S2 in File S1. The apparent odds ratio rises monotonically as samples are taken from greater extreme of the risk score, and also rises as the variance explained by this risk score increases. Larger odds ratios show proportionally larger increases, and more prevalent diseases also show a stronger effect.

Notably, the increase in apparent odds ratio is largely independent of risk allele frequency. This suggests that prioritisation will be equally effective in increasing power across the frequency spectrum, adding power to rare and low frequency designs as well as common variant fine-mapping and replication designs. Finally, selection of controls from the general population (i.e. when a proportion 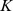 of them have the disease) has no effect on the allele frequency. This suggests that prioritization of controls will only increase power if those controls have been screened for the disease. This is usually the case for the control cohorts used to study most common diseases such as type 2 diabetes, but usually not for those used for rarer diseases such as Crohn's disease.

### Power calculations in unrelated individuals

**Figure 2:**
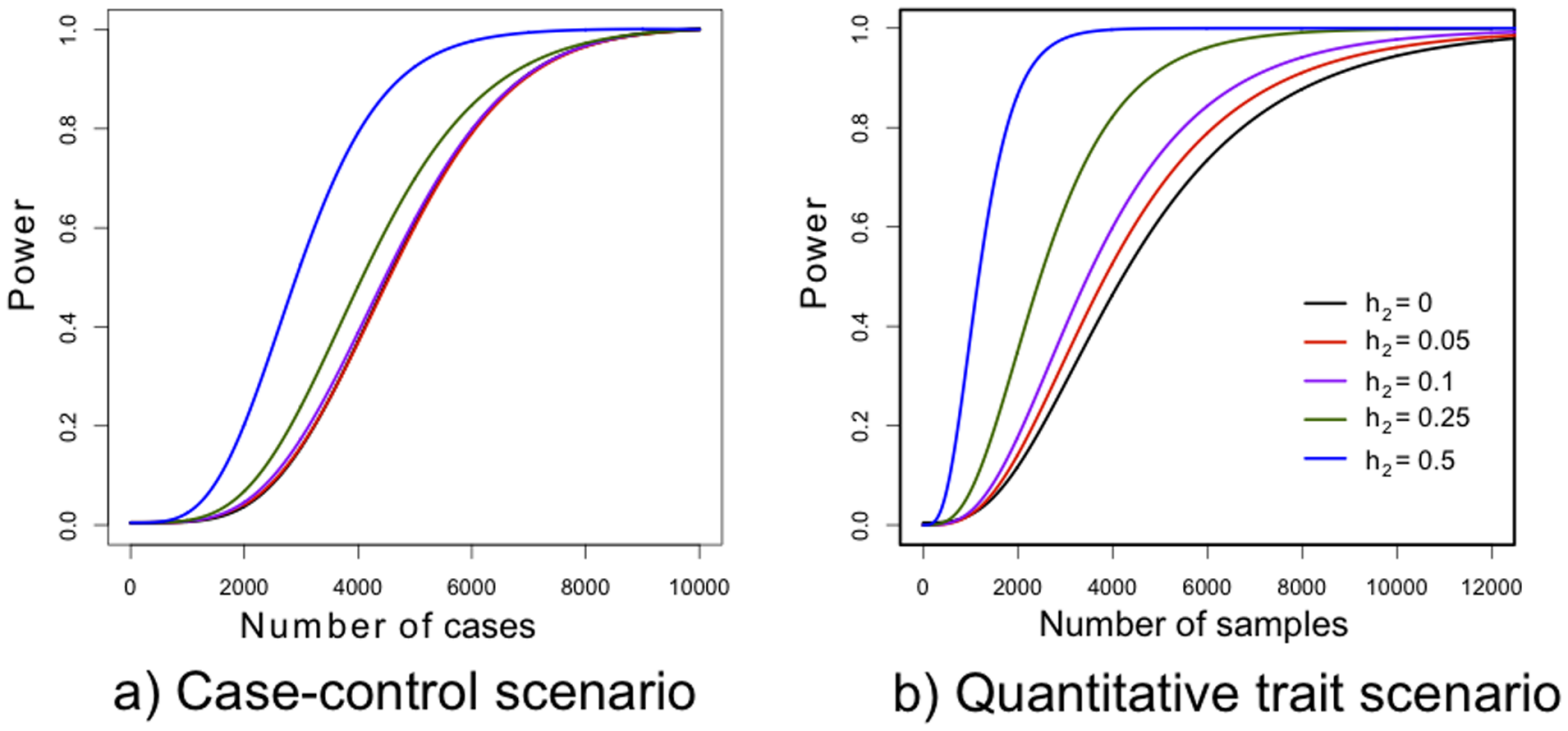
Improvement in power curves gained by prioritising samples based on genetic risk scores with different predictive powers. The color of the line represents the proportion of total variance capture by the risk score, with the black line representing a random (i.e. non-prioritised) selection of samples. a) A case-control scenario for a disease with 1% prevalence. The total cohort size for prioritisation is 10,000 cases and an equal number of controls, and we measured power to detect a risk allele with an odds ratio of 2 and a frequency of 1% at genome-wide significance. b) A quantitative trait scenario. The total cohort size is 100,000, and we measured power to detect an allele with 1% frequency that increased a normally-distributed quantitative trait by 0.2 standard deviations. Power is calculated using the trend test power equations given by Freidlin *et al* [36].

To see the impact of this selection strategy on the power of an experiment, consider a collection of 10,000 cases of a disease with a prevalence of 1% (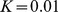) along with a collection of 10,000 healthy controls. We wish to pick *N* cases and *N* controls for whole-genome sequencing with the goal of identifying, at genome-wide significance (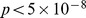), a 1% risk allele with an odds ratio of 2 (a typical design, though optimal case-control ratio and p-value thresholds for rare variant studies are debatable). We compare random selections of cases and controls to selections informed by known risk loci as described above. Figure 2a shows the increased power conferred by genetic predictors explaining 5–50% of liability-scale variance. Since GWAS explain <10% of variance of most complex diseases, the power gained by this approach would be marginal in most realistic circumstances. Nonetheless, complex diseases with particularly good GWAS predictors, such as type 1 diabetes or age related macular degeneration, could see substantial power gains.

Because the sampling approach works best given a large pool of individuals to draw from, we next considered a large population cohort of 100,000 individuals measured for some continuous trait. In this case we evaluated the power to detect, again at genome-wide significance, a 1% risk variant with a *β* value of 0.2. *N* individuals from each of the two extremes of 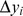 were selected and treated as cases and controls and compared to drawing from the extremes of 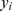. Figure 2b shows that improvement in power from the prediction-informed selection is relatively large even when the prediction explains <10% of variance.

### Testing a hypothetical experiment using real data

The above power calculations are based on assumptions about effect size, predictive capacity and allele frequencies of risk variants in hypothetical studies. To demonstrate that prioritisation could add power in a real world scenario, we used real data to simulate a hypothetical but realistic low-frequency disease association experiment.

In our hypothetical design we perform targeted sequencing on candidate genes in 1,000 cases and 1,000 controls selected from a large cohort, then select the 50 SNPs with the lowest p-values and genotype them in the rest of the cohort. We assume that we would discover 1,000 coding variants (a typical number if we sequenced around 60 genes [18]), of which 5 are truly associated and 995 are not.

We based our simulations on 19,761 Crohn's disease cases and 28,999 controls genotyped by the International IBD Genetics Consortium's (IIBDGC) Immunochip project [19]. For the truly associated variants, we used five low-frequency coding variants discovered by Rivas *et al* [20] which were included on the Immunochip array and reached genome-wide significance (p<5×10^−8^) in the complete dataset. To perform risk prediction and prioritisation we used odds ratios and frequencies from the 166 Crohn's disease risk variants from the latest IIBDGC Immunochip analysis [19].

We simulated 1,000 studies from these data. The controls were selected at random (as they were population controls and thus selection would not grant extra power), and the cases were either selected at random or were selected from the bottom quartile of Crohn's disease risk. We selected from the bottom quartile, rather than a more extreme selection, in order to allow room to resample and thus assess the significance of the results. On average the prioritised study discovered significantly more of the Rivas *et al* variants than the non-prioritised study (1.64 vs 1.48, rank sum p-value = 0.001). The improvement is modest but real, with the prioritisation increasing the probability of discovering 3 or more of the Rivas *et al* variants from 12% to 19%, demonstrating that our method can measurably increase power in the design of real experiments.

### Power simulations in families

The study of families with multiple individuals affected by a complex disease is a potentially powerful means of identifying alleles which are rare in the population, but confer substantial risk. Historical family studies have produced a large number of suggestive linkage peaks, but few that were successfully replicated [21], [22]. Some of these failed replications may be caused by artefacts or statistical noise, but at least some are likely to be true signals. GWAS have suggested that complex diseases are influenced by hundreds of separate loci, which might mean that families sharing the same clinical diagnosis in fact harbor mutations in a diverse set of genes. For instance, it has been estimated that between 1 and 3% of diabetes cases are caused by penetrant mutations, but over 20 genes carrying such mutations have been identified to date [23]. Such locus heterogeneity would seriously reduce the power of cross-family linkage analysis, but could be overcome by combining within-family linkage information with complete sequence data [24].

**Figure 3:**
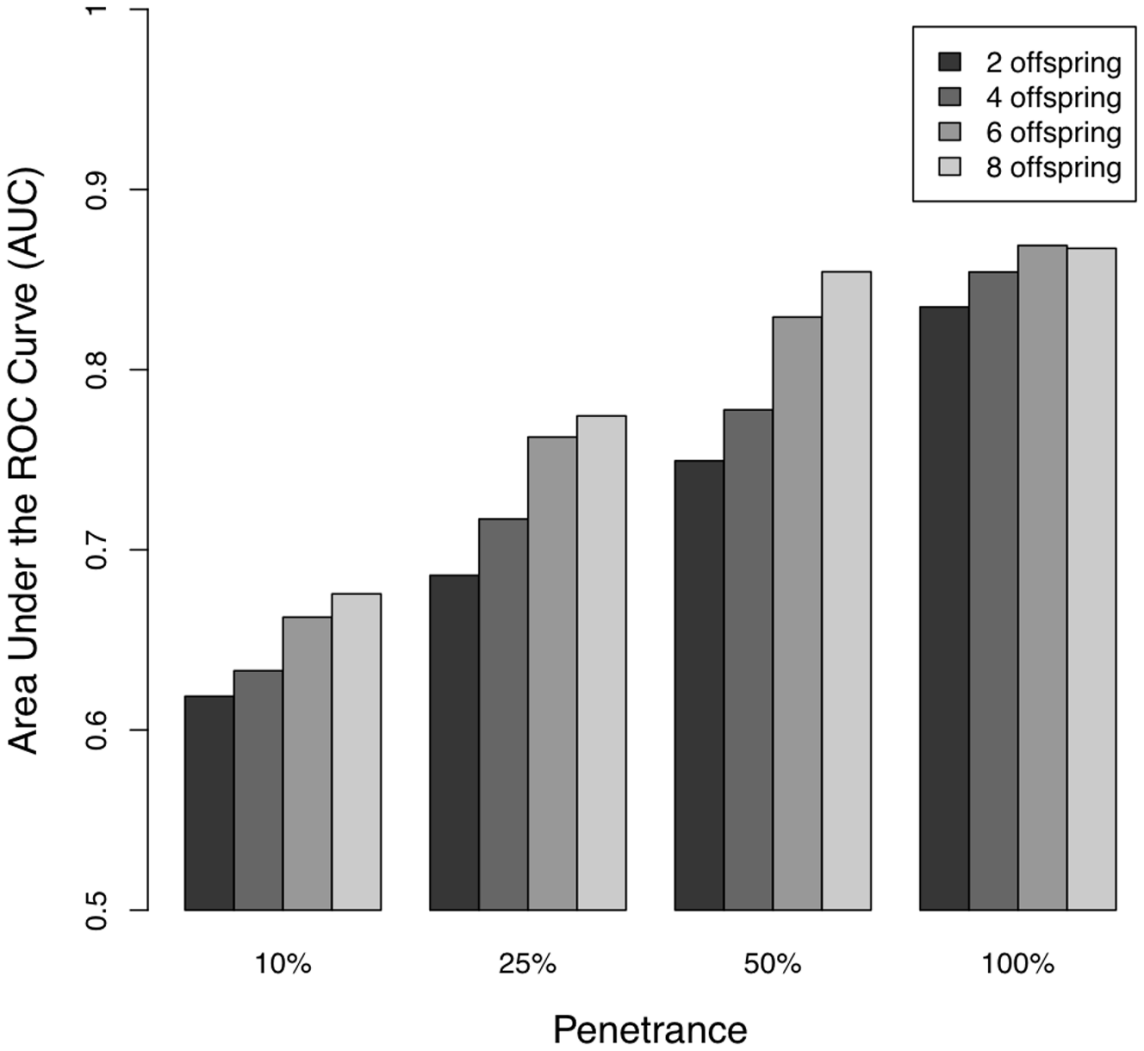
Ability to predict the presence of a high-penetrance mutation (measured by AUC) in multiplex families using a polygenic risk score. We assume a disease with a prevalence of 1% and a heritability 50%, and a genetic risk score that captures 12.5% of variance. All families have three affected individuals, and the AUC is shown for families of different total size and dominant mutations of varying penetrance.

In order to most efficiently design such studies, we have extended our risk prediction approach to families both in order to distinguish between those with the highest burden of unexplained disease and those for whom disease burden is largely or completely explained by known GWAS loci. Assume that a given family has *N* members, of whom *y* are affected. We wish to select families for which *y* is significantly larger than what would be expected given the observed genotypes, *G*, i.e. those that minimize:
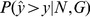
(1)To evaluate the power of this approach for prioritizing families for sequencing experiments, consider two families: both are subject to polygenic risk for a disease but only one contains an additional high penetrance dominant mutation. We would like to be able to prioritize the latter family for the type of family sequencing experiment described above. To evaluate the ability of our method to identify families containing such high penetrance mutations we simulated nuclear families with between 2 and 8 offspring, where three total family members were affected by a disease having 1% prevalence and heritability of 50% (these values correspond approximately to immune mediated diseases such as Crohn's disease). Half the families contained a dominant mutation with a penetrance from 10–100%, and the other half arose simply from polygenic risk and chance. For each family, we computed the value of equation (1) based on a GWAS risk predictor explaining 25% of heritability (again by analogy to Crohn's disease [25]). Figure 3 shows the area under the ROC curve (AUC), which in this instance can be interpreted as the probability of correctly distinguishing between one family with a penetrant mutation and one without. For a low-penetrance mutation in a small family AUC is only ∼0.6, but for a medium-penetrance mutation in a large family, AUC is ∼0.85, which would provide a substantial advantage over simply selecting the family with the largest number of affected individuals.

### A new algorithm for risk prediction in partially genotyped families

If the genotypes *G* are known for all family members then calculating equation (1) is relatively simple. However, most family based experiments will not generate genotype data across all members of the pedigree for a variety of reasons, including cost, DNA availability, consent, or death. Performing risk prediction in the family without accounting for individuals without genotypes will introduce bias (as in general a higher proportion of cases are genotyped than unaffected relatives) as well as noise (as this sampling bias will vary from family to family).

A solution to this issue is to sample genotypes for the ungenotyped individuals conditional on the genotyped ones, and perform risk prediction on these sampled genotypes. We have produced an efficient algorithm, based on an application of a modified Inside-Outside algorithm [26], to allow this sampling to be performed efficiently even on very large pedigrees. We have used this algorithm to perform risk prediction on partially genotyped extended pedigrees with that included over 800 individuals (data not shown). Details of the algorithm are given in the Methods section below, and in the Appendix.

## Discussion

We have presented a new method for genetic risk prediction using known GWAS-type risk variants to inform the design of sequencing studies aimed at finding low-frequency or rare risk alleles. We have shown via simulation that this approach can increase the power of sequencing studies in both case/control and family settings. The power increase is greatest when it is possible to sample from a large pool of potential individuals or families for sequencing (e.g. for the study of lipid levels in large cohorts of healthy individuals [27]), and when a substantial fraction of heritability is explained by known GWAS loci (e.g. type 1 diabetes [28], age related macular degeneration [29] or required warfarin dose [30]). We have here assumed that the risk score and the risk variant act independently, but the presence of gene-gene interaction may alter the power of this approach. For instance, the increase in power due to prioritisation will be greater if the effect size of the locus being tested decreases as the risk score increases, as is known to be the case between HLA risk and other GWAS variants in type 1 diabetes [31].

A major advantage of this approach is that it will become more effective with time; as more genetic risk factors are discovered, new prediction algorithms are developed [32] and estimates of effect sizes are refined [33], the accuracy of prediction will increase. By sequentially incorporating risk prediction into our study designs, we can utilize each new discovery to increase our power in future research. We also note that while we have exclusively used genetic examples, the same framework can incorporate environmental risk factors (as discussed by Guey et al [14]). This allows a straightforward improvement of the predictors, and thus the efficacy of prioritisation. It also intriguingly suggests the possibility of using genetics to inform studies of environmental and epigenetic risk. For example, a family with a higher-than-expected incidence of disease given their genotypes at known risk loci is more likely to harbor a penetrant mutation, but is also more likely to have been exposed to a dietary risk factor or risk-increasing infection. This suggests a broad range of potential uses of risk prediction in study design, both from genes to environment and environment to genes.

Software implementing the methods in this paper, as well as documentation for their use in prioritisation, is available from CRAN as an R package named Mangrove.

## Methods and Models

### Risk prediction in unrelated individuals

To predict a quantitative phenotype from genotypes at a known set of GWAS loci, consider a matrix of genotypes, *G*, with elements 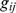 for locus *i* and individual *j*, and a vector of standardised effect sizes 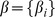.

The quantitative trait predictions, 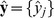 are calculated via a linear model:
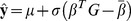
(2)where 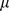 and 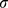 are the population mean and standard deviation of the quantitative trait, and 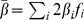 is a normalising constant accounting for 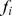, the allele frequency.

For binary traits, we use a logistic link function to produce probabilities of disease status:
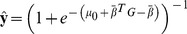
(3)Where here 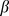 is a vector of log odds ratios, and 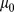 is a function of 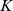, representing the prevalence (for incidence prediction) or life-time risk (for prospective prediction) of the disease.

### Risk prediction in partially genotyped families

If the genotypes 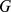 are known for all family members then disease probabilities for each individual can be calculated as in equation (3), and then used to calculate equation (1) by sampling. However, most family based experiments will not generate genotype data across all members. A solution is to sample disease status as in the complete information case, conditional on a set of of unobserved genotypes 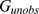 that are themselves sampled from the conditional distribution:
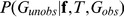
(4)Where 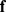 is the population allele frequency, 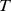 is the family structure, and 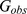 are the known genotypes. Sampling from this distribution is not trivial, but is possible via a modified Inside-Outside algorithm [26] (itself a generalisation of the forward-backwards algorithm used in Hidden Markov Models [34]). The Inside-Outside is used for inference on tree-like data structures, and has been applied to certain multiple sequence alignment problems [35]. Here, we instead use Inside-Outside to sample from the posterior distribution of genotypes across a family. Briefly, we decompose the marginal genotype posteriors into inside and outside probabilities, similar to the forward and backward probabilities from an HMM. The inside probability accounts for information from each individual and their descendants, whereas the outside probability accounts for the individual's other relatives (including ancestors, siblings and cousins).

These values can be computed recursively via the standard Inside-Outside approach (Appendix B.1 in File S1), which enables the sampling of one individual's genotypes. When sampling an entire family, however, we must sample down the tree from the root, with each individual's genotypes conditioned on their parents' sampled genotypes. We accomplish this by modifying the outside probability to include parental genotypes (Appendix B.2 in File S1). We describe some additional considerations required for the application of Inside-Outside to family trees in Appendix C in File S1.

Note that in this paper we only consider genetic risk that is explained by genotypes in the matrix 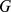. In reality, there is additional genetic risk due to undiscovered risk variants. While this does not contribute to the selection scheme, and is thus not relevant here, the approach outlined above can easily include samples from a unobserved normally distirbuted polygenetic risk score with a covariance matrix dependent on 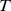.

### Implementation

The methods discussed in this paper are implemented in the R package Mangrove, available from CRAN: http://cran.r-project.org/web/packages/Mangrove/index.html

## Supporting Information

File S1 **Appendices A, B and C and Figures S1 and S2.** Appendix A describes methods for predicting allele frequencies after prioritisation and appendices B and C describe the modified Inside-Outside algorithm and its application to family data. Figure S1 shows the distribution of Δy under different disease prevalence and risk score predictive powers, and Figure S2 shows the effect of disease prevalence and allele frequency on the effectiveness of prioritisation.
(PDF)
