# Supplementary material for: Using Genetic Prediction from Known Complex Disease Loci to Guide the Design of Next-Generation Sequencing Experiments
Source: PLoS One. 2013 Oct 18;8(10):e76328. doi: 10.1371/journal.pone.0076328 (PMC3799779; doi:10.1371/journal.pone.0076328)
Supplement: File S1 — Appendices A, B and C and Figures S1 and S2. Appendix A describes methods for predicting allele frequencies after prioritisation and appendices B and C describe the modified Inside-Outside algorithm and its application to family data. Figure S1 shows the distribution of Δy under different disease prevalence and risk score predictive powers, and Figure S2 shows the effect of disease prevalence and allele frequency on the effectiveness of prioritisation. (PDF) [file pone.0076328.s001.pdf]

Appendices for "Using genetic prediction from known complex disease loci to guide the design of next-generation sequencing experiments"

Luke Jostins,[1,2,*] Adam P. Levine,[3] Jeffrey C. Barrett[2]

[1]Wellcome Trust Centre for Human Genetics, Univeristy of Oxford, Roosevelt Drive, Oxford OX3 7BN

[2]Medical Genomics, Wellcome Trust Sanger Institute, Wellcome Trust Genome Campus, Hinxton, Cambridge CB10 1HH, UK

[3]Division of Medicine, University College London, 5 University Street, London WC1E 6JF, UK

*Contact: lj4@well.ox.ac.uk

A Modelling selection based on risk scores

A.1 Binary traits

Assume that a disease has a prevalence K, and has a risk score that explains a proportion of variance h_p^2. We model the value of this risk score for individual i as normally distributed, i.e. $\eta_i \sim N(\mu, \sigma^2)$. The disease probability, conditional on this risk score, is then given by a logistic link function

$$P(D|\eta_i) = \hat{y}_i(\eta_i) = (1 + \exp(-\eta_i))^{-1} \tag{1}$$

The distributions of η_i in cases and controls are given by

$$\begin{aligned} f(\eta_i|D) &= \frac{P(D|\eta_i)f(\eta_i)}{P(D)} && (2) \\ &= \frac{\hat{y}_i(\eta_i)}{K} f(\eta_i) && (3) \\ f(\eta_i|!D) &= \frac{1 - \hat{y}_i(\eta_i)}{1 - K} f(\eta_i) && (4) \end{aligned}$$

where $f(\eta_i) = \frac{1}{\sigma}\phi\left(\frac{\eta_i - \mu}{\sigma}\right)$.

To fit this risk score model, we use the equations given by Wray *et al*[10] to convert h_p^2 into an equivalent λ_s value. We then numerically fit μ and σ to give $E[\hat{y}_i] = K$ and $E[\hat{y}_i\hat{y}_j]/K^2 = \lambda_s$, where $cor(\eta_i, \eta_j) = 0.5$ (i.e. the correlated risk scores of siblings i and j).

We can expand this model to include an additional risk variant with frequency q and odds ratio r, assumed to be in Hardy-Weinberg equilibrium, and independent of (i.e. not included in or interacting with) the risk score η_i. If an individual's genotype is given by $g_i \in (0, 1, 2)$, the link function becomes

$$P(D|\eta_i, g_i) = \hat{y}'_i(\eta_i, g_i) = (1 + \exp(-\eta_i - \beta_0 - \beta_1 g_i))^{-1} \tag{5}$$

The joint distribution of η_i and g_i in cases is given by

$$f(g_i, \eta_i|D) = \frac{\hat{y}'_i}{K} P(g_i) f(\eta_i) \tag{6}$$

where $P(g_i) = \binom{2}{g_i} q^{g_i}(1-q)^{1-g_i}$. Equally

$$f(\eta_i, g_i|!D) = \frac{1-\hat{y}_i'}{1-K}P(g_i)f(\eta_i) \quad (7)$$

The marginal genotype distributions are then given by

$$P(g_i,|D) = \int_{-\infty}^{\infty} f(g_i,\eta_i|D)d\eta_i \quad (8)$$

$$P(g_i,|!D) = \int_{-\infty}^{\infty} f(g_i,\eta_i|!D)d\eta_i \quad (9)$$

We then numerically fit β_0 and β_1 to give $E[\hat{y}_i'] = K$ and $\frac{E[g_i|D]}{1-E[g_i|D]}\frac{1-E[g_i|!D]}{E[g_i|!D]} = r$.

Assume that we select all cases with $\eta_i < T_{case}$ (equivalent to $\Delta y > (1+\exp(-T_{case}))^{-1}$). The genotype distribution in this group will then be

$$P(g_i|\eta_i < T_{case}, D) = \frac{P(g_i,\eta_i < T_{case}|D)}{P(\eta_i < T_{case}|D)} \quad (10)$$

$$= \frac{\int_{-\infty}^{T_{case}} f(g_i,\eta_i|D)d\eta_i}{\int_{-\infty}^{T_{case}} f(\eta_i|D)d\eta_i} \quad (11)$$

$$= P(g_i)\frac{\int_{-\infty}^{T_{case}} \hat{y}_i'(\eta_i,g_i)f(\eta_i)d\eta_i}{\int_{-\infty}^{T_{case}} \hat{y}_i(\eta_i)f(\eta_i)d\eta_i} \quad (12)$$

and, likewise, taking only controls with a risk score $\eta_i > T_{control}$

$$P(g_i|\eta_i > T_{control}, D) = P(g_i)\frac{\int_{T_{control}}^{\infty}(1-\hat{y}_i'(\eta_i,g_i))f(\eta_i)d\eta_i}{\int_{T_{control}}^{\infty}(1-\hat{y}_i(\eta_i))f(\eta_i)d\eta_i} \quad (13)$$

We can then calculate the odds ratio for a given pair of cut-offs T_{case} and $T_{control}$ by calculating $E[g_i|D,\eta_i < T_{case}]$ and $E[g_i|!D,\eta_i > T_{case}]$.

Note that if controls are population controls, i.e. a proportion K of them have the disease, g_i and η_i are independent, and thus $E[g_i|Control,\eta_i > T_{control}] = E[g_i] = q$. This means that selecting population controls does not change the risk allele frequency, and thus does not change the estimation of the odds ratio.

A.2 Continuous traits

When modeling the effect of a variant with genotype g, with effect size β, on a standard normally distributed quantitative trait we use the standard equation for linear regression:

$$y = g\beta - \bar{\beta} + e \tag{14}$$

where $var(y) = 1$, $var(g\beta) = 2q(1-q)\beta^2$ and thus $var(e) = 1 - 2q(1-q)\beta^2$. The probability of an individual having y greater than some threshold T, conditional on g is:

$$\begin{aligned} P(y > T|g) &= P(g\beta - \bar{\beta} + e > T) && (15)\\ &= P(e > T - g\beta + \bar{\beta}) && (16)\\ &= \Phi\left(-\frac{T - g\beta + \bar{\beta}}{\sqrt{1 - 2q(1-q)\beta^2}}\right) && (17) \end{aligned}$$

therefore

$$P(g|y > T) = \frac{P(y > T|g)P(g)}{\sum_{g'} P(y > T|g')P(g')} \tag{18}$$

If we split the error term into $e = \hat{y} + e'$, where $\hat{y}$ is a prediction of y with variance h_p^2 we have

$$y = g\beta - \bar{\beta} + \hat{y} + e' \tag{19}$$

with $var(e') = 1 - h_p^2 - 2q(1-q)\beta^2$. We can then define the difference between predicted and actual phenotype as

$$\Delta y = y - \hat{y} = g\beta - \bar{\beta} + e' \tag{20}$$

and thus

$$P(\Delta y > T|g) = \Phi\left(-\frac{T - g\beta + \bar{\beta}}{\sqrt{1 - h_p^2 - 2q(1-q)\beta^2}}\right) \tag{21}$$

We can then apply equation 18 to give $P(g|\Delta y > T)$.

B The Inside-Outside algorithm on directed trees

B.1 Description of the basic algorithm

Definitions

The Inside-Outside algorithm is a generalisation of the Forward Backward algorithm, originally designed to extend parameter estimation from Hidden Markov Models[8] to stochastic context-free grammars[1]. Here we reformulate the Inside Outside algorithm as a method of performing parameter estimation and sampling on a directed tree.

By a directed tree we mean a directed acyclic graph with all edges directed away from the root (i.e. towards the leaves). We will denote nodes by subscripts i, j, k. Each node i may have a parent p_i, offspring o_i and/or siblings s_i. The node without parents is called the "root node" or "root", and a node without children is called a "leaf node" or "leaf".

Each node i has an associated emission d_i (for instance, an observed genotype), as well as a hidden state x_i (e.g. an inferred genotype) with statespace S_i. The values of hidden states will be denoted a, b, c etc, e.g. $(x_i = a)$ denotes that node i has hidden state value a.

The tree defines a graphical model that specifies the probability density functions for all the variables (hidden states and emissions) as conditional probabilities. Specifically, the probability density function of emission d_i is specified conditional on hidden state x_i taking on value a by the likelihood:

$$L_i(a) = P(d_i|x_i = a) \tag{22}$$

Note that that we assume $d_i \perp (d_j, x_j) \mid x_i : \forall j \neq i$), i.e. that the emission for node i is independent of all other emissions and hidden states, conditional on the hidden state of node i.

The probability density function for a non-root hidden state x_i taking on value b is specified conditional on the parent's hidden state x_{p_i} taking on value a by the transition probability:

$$T_i(b|a) = P(x_i = b|x_{p_i} = a) \tag{23}$$

Note the assumption $x_i \perp x_j \mid x_{p_i} : \forall j \neq i, p_i$.

The probability distribution of the hidden state associated with the root x_{root} is given by the root prior:

$$\pi(a) = P(x_{root} = a) \tag{24}$$

We will refer to all emissions associated with nodes descended from node i as D_i, and all emissions not associated with node i or its descendants as $D_{!i}$. Note that these can both be expressed recursively:

$$D_i = \{d_i, D_{o_i}\} \tag{25}$$

for non-leaves and $D_i = d_i$ for leaves, and

$$D_{!i} = \{D_{s_i}, D_{!p_i}, d_{p_i}\} \tag{26}$$

for non-roots and $D_{!i} = \emptyset$ for the root. All emissions associated with all nodes can be expressed as D, and $D = \{D_i, D_{!i}\}$ for any i.

We will use the Inside-Outside algorithm to deduce the probability density functions of each hidden state (x_i) conditional on observed emissions associated with all nodes (D).

The Inside Probability

The inside probability $\alpha_i(a)$ is defined as the probability of observing the emission associated with node i and all its descendants, given that the hidden state x_i takes on value a:

$$\alpha_i(a) = P(D_i | x_i = a) \tag{27}$$

For leaves, $D_i = d_i$, and hence $\alpha_i(a) = L_i(a)$. For non-leaves:

$$\begin{aligned}
\alpha_i(a) &= P(D_i|x_i = a) \\
&= P(d_i|x_i = a) \prod_{j \in o_i} P(D_j|x_i = a) \\
&= P(d_i|x_i = a) \prod_{j \in o_i} \sum_{b \in S_j} P(D_j|x_i = b) P(x_j = b|x_i = a) \\
&= L_i(a) \prod_{j \in o_i} \sum_{b \in S_j} \alpha_j(b) T_j(b|a)
\end{aligned} \tag{28}$$

Because we require the inside probabilities of all offspring of a node to calculate its own inside

probability we calculate the inside probabilities first for the leaves, and then propagate them recursively up the tree. The overall likelihood of all emissions D is:

$$P(D) = \sum_{a \in S_{root}} \alpha_{root}(a)\pi(a) \tag{29}$$

The Outside Probability

The outside probability $\beta_i(a)$ is defined as the joint probability of observing emissions not associated with node i and its descendants, and the node i having hidden state $x_i = a$:

$$\beta_i(a) = P(D_{!i}, x_i = a) \tag{30}$$

For the root node, $D_{!i} = \emptyset$, so $\beta_{root}(a) = P(x_{root} = a) = \pi(a)$. For non-root nodes, we can calculate the outside probability recursively:

$$\begin{aligned}
\beta_i(a) &= P(D_{!i}, x_i = a) \\
&= \sum_{c \in S_{p_i}} P(x_{p_i} = c, x_i = a, D_{!i}) \\
&= \sum_{c \in S_{p_i}} P(x_{p_i} = c, x_i = a, D_{!p_i}) P(d_i | x_{p_i} = c) \prod_{j \in s_i} P(D_j | x_{p_i} = c) \\
&= \sum_{c \in S_{p_i}} P(x_{p_i} = c, D_{!p_i}) P(x_i = a | x_{p_i} = c) P(d_i | x_{p_i} = c) \prod_{j \in s_i} \sum_{b \in S_j} P(D_j | x_j = b) P(x_j = b | x_{p_i} = c) \\
&= \sum_{c \in S_{p_i}} \beta_{p_i}(c) T_i(a|c) L_{p_i}(c) \prod_{j \in s_i} \sum_{b \in S_j} \alpha_j(b) T_j(b|c)
\end{aligned} \tag{31}$$

The outside probability for each node requires the outside probability of the node's parent. We thus calculate it first for the root, and then propagate recursively down the tree. The outside probabilities are also dependent on the inside probabilities, which are therefore calculated first.

B.2 Conditional sampling across the tree

We can calculate the posterior distribution of hidden state x_i conditional on all emissions D in terms of the inside and outside probabilities:

$$P(x_i = a | D) = \frac{\alpha_i(a)\beta_i(a)}{P(D)} \tag{32}$$

We can sample from this posterior distribution for each node. However, this approach cannot

jointly sample hidden states across the entire tree. To do this we need to propagate sampled states down the tree, starting with the root. The hidden state for the root can be sampled from the posterior:

$$P(x_{root} = a|D) = \frac{\alpha_{root}(a)\pi(a)}{P(D)} \tag{33}$$

To sample non-roots, we must first calculate the partial outside variable, which includes the hidden state c of the parent, and can be calculated as:

$$\begin{aligned}\beta_i^p(a,c) &= P(x_i = a, x_{p_i} = c, D_{!i}) \\ &= \beta_{p_i}(c)T_i(a|c)L_{p_i}(c)\prod_{j\in s_i}\sum_{b\in S_j}\alpha_j(b)T_{jp_i}(b|c)\end{aligned} \tag{34}$$

The hidden state of node i can then be sampled from the posterior conditional on the sampled hidden state of the parent c:

$$P(x_i = a|D, x_{p_i} = c) = \frac{\beta_i^p(a,c)\alpha_i(a)}{\sum_{a\in S_i}\beta_i^p(a,c)\alpha_i(a)} \tag{35}$$

Like the calculation of the outside probabilities, the samples are propagated down the tree.

C Application of the Inside-Outside algorithm to family trees

A family tree is not strictly a directed tree, due to the addition of new founders (via marriage) in each generation. However, we can make most family trees into directed trees by treating parent couples as a single node, consisting of a founder and a non-founder individual. The root node of this directed family tree consists of the top pair of founders.

`Mangrove` will automatically carry out this conversion from family tree to directed tree for families with only one founder-founder couple. However, in theory any family relationships that do not include inbreeding (i.e. all families that take the form of a polytree) can be modelled as a directed tree by choosing an arbitrary pair of founders as the root, and reversing the direction of all connections originating from parent couples that are not descended from this root node. The genotypes of these parents couples are then given conditional on their children's genotypes by the

reverse transition matrix $T_{p_i}(a|b) = T_i(b|a)\frac{P(x_{p_i})}{P(x_i)}$, where $P(x_{p_i})$ and $P(x_i)$ are given by the allele frequency. However, this is not currently implemented in `Mangrove`. at this time.

We use the Inside-Outside algorithm to sample unobserved genotypes conditional on all other genotypes for a single biallelic polymorphism with allele frequency f (although this is readily generalised to an arbitrary number of independent polymorphisms). We model individuals as nodes, and genotypes as a hidden state for each node. For non-parent couples the state-space is:

$$x_i \in S_i = \{AA, AB, BB\} \tag{36}$$

and for parent couples it is

$$x_i = (x_i^f, x_i^{nf}) \in \{AA, AB, BB\}^2 \tag{37}$$

where x_i^f is the founder's genotype state and x_i^{nf} is the non-founder's genotype.

Genotype calls for each individual are modeled as emissions, and we assume that these genotypes are certain and thus for genotyped individuals x_i and d_i are identical (though genotype error can be included by modifying the likelihoods below). Genotypes can also be missing (N). Thus the emission for a non-parent couple node is:

$$d_i = g_i \tag{38}$$

and for parent couples is

$$d_i = \{g_i^f, g_i^{nf}\} \tag{39}$$

Likelihoods for non-parent couples are:

$$L_i(a) = \begin{cases} 1 & \text{if } a = g_i \text{ or } g_i = N; \\ 0 & \text{otherwise.} \end{cases} \tag{40}$$

and for parent couples are

$$L_i(a) = \begin{cases} 1 & \text{if } a_i^f = g_i^f \text{ and } a_i^{nf} = g_i^{nf}; \\ 1 & \text{if } a_i^f = g_i^f \text{ and } g_i^{nf} = N \text{ or } a_i^{nf} = g_i^{nf} \text{ and } g_i^f = N; \\ 1 & \text{if } g_i^f = g_i^{nf} = N; \\ 0 & \text{otherwise.} \end{cases} \tag{41}$$

Transitions can only occur from a parent couple to a non-parent couple, or from a parent couple to a parent couple. For a parent couple to a non-parent couple, transmission is simple Mendelian inheritance:

$$T_{ij}(a|b) = P(C = a|P1 = b^f, P2 = b^{nf}) \tag{42}$$

where C is the child's genotype, and $P1$ and $P2$ are parental genotypes. For parent couple to parent couple transmission, we need to include the probability density on the founder genotype

$$T_i(a|b) = P(C = a^{nf}|P1 = b^f, P2 = b^{nf})P(a^f|f) \tag{43}$$

where $P(a^f|f)$ is the population frequency of the founder's genotype, assuming Hardy-Weinberg equilibrium. Finally, the prior on the root node is given by the population frequency:

$$\pi(a) = P(a^{f_1}|f)P(a^{f_2}|f) \tag{44}$$

where a^{f_1} and a^{f_2} are the two founder individuals that make up the root node.

Using this formulation, marginal posteriors can be calculated for each unobserved genotype, and joint genotypes for the entire family can be sampled from the joint posterior distribution.

C.1 Comparison of the Inside-Outside algorithm to other pedigree techniques

Many other methods have been developed to calculate likelihoods, marginal posteriors and samples from pedigree data. Some are similar to the Inside-Outside approach described above:

- The calculation of the Inside probabilities is equivalent to the calculation of the likelihood in the original Elston-Steward algorithm[4]
- The Inside and Outside probabilities are equivalent to the Upper and Lower probabilities in Cannings et al[2], and to R* and R+ in Cannings et al[3]. The calculation of these parameters

is equivalent to the nuclear family peeling described in Section 4.11 of Cannings et al [3].

- Many algorithms exist for sampling genotypes from a complex pedigree [7;5;9]. However, these methods tend to be relatively inefficient as they need to handle the complex interdependencies in the pedigree, either by storing large intermediate results or using approximate iterative techniques such as MCMC.

- Our method is similar to that of Fernando et al [6], who develop a method for calculating exact posterior genotype distributions across a non-looped pedigree. They also use a recursive method that avoids recomputing intermediate results, and a generalization of their approach to joint sampling across the pedigree would likely produce an algorithm equivalent to ours.

The main disadvantage of our method compared to existing methods is that it assumes unlinked genotypes in a non-looped pedigree. However, the main advantage is that it allows precomputation of all conditional genotype probabilities. As a result, once α, β and β^p have been calculated, sampling of genotypes can be carried out very rapidly, allowing thousands of samples to be carried out in pedigrees with thousands of individuals and hundreds of markers.

References

[1] J.K. Baker. Trainable grammars for speech recognition. *The Journal of the Acoustical Society of America*, 65:S132, 1979.

[2] C Cannings, EA Thompson, and HH Skolnick. The recursive derivation of likelihoods on complex pedigrees. *Advances in Applied Probability*, 8(4):622–625, 1976.

[3] C Cannings, EA Thompson, and MH Skolnick. Probability functions on complex pedigrees. *Advances in Applied Probability*, pages 26–61, 1978.

[4] R. C. Elston and J. Stewart. A general model for the genetic analysis of pedigree data. *Hum. Hered.*, 21(6):523–542, 1971.

[5] S. A. Fernandez, R. L. Fernando, B. Guldbrandtsen, C. Stricker, M. Schelling, and A. L. Carriquiry. Irreducibility and efficiency of ESIP to sample marker genotypes in large pedigrees with loops. *Genet. Sel. Evol.*, 34(5):537–555, 2002.

[6] RL Fernando, C Stricker, and RC Elston. An efficient algorithm to compute the posterior genotypic distribution for every member of a pedigree without loops. *Theoretical and Applied Genetics*, 87(1-2):89–93, 1993.

[7] J. Ott. Computer-simulation methods in human linkage analysis. *Proc. Natl. Acad. Sci. U.S.A.*, 86(11):4175–4178, Jun 1989.

[8] L.R. Rabiner. A tutorial on hidden markov models and selected applications in speech recognition. *Proceedings of the IEEE*, 77(2):257–286, 1989.

[9] L. R. Totir, R. L. Fernando, and J. Abraham. An efficient algorithm to compute marginal posterior genotype probabilities for every member of a pedigree with loops. *Genet. Sel. Evol.*, 41:52, 2009.

[10] N.R. Wray, J. Yang, M.E. Goddard, and P.M. Visscher. The genetic interpretation of area under the ROC curve in genomic profiling. *PLoS Genet.*, 6(2):e1000864, 2010.

Supplementary figures

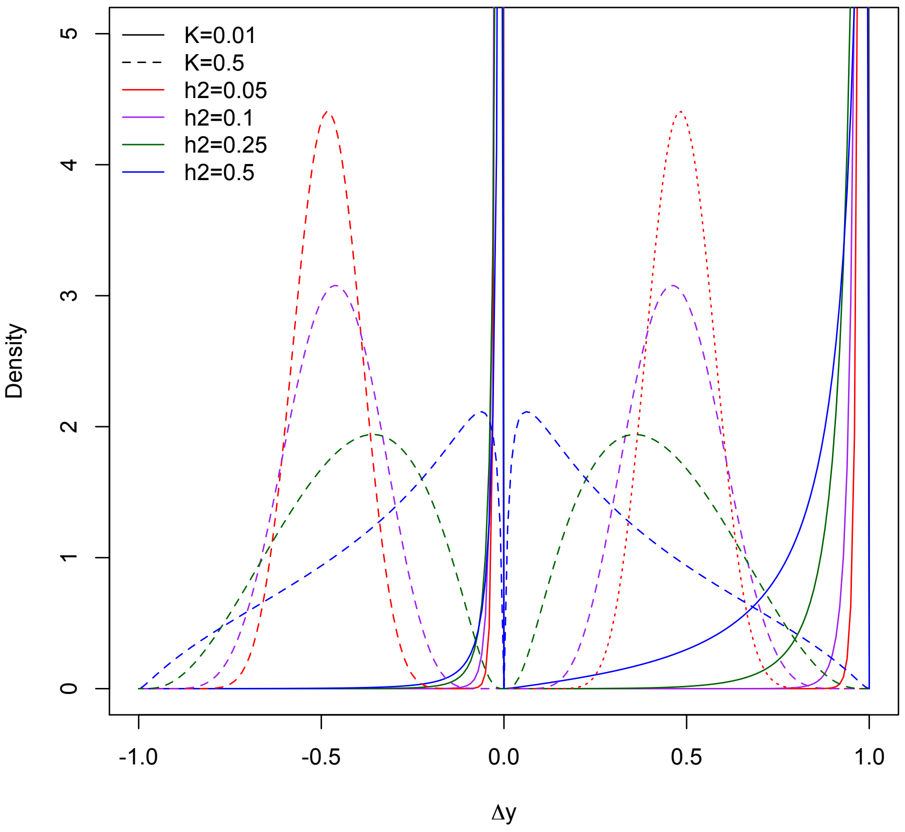

Figure S1: The distribution of Δy in an uncommon ($K = 0.01$) and a very common ($K = 0.5$) disease, for different values of the heritability h_p^2.

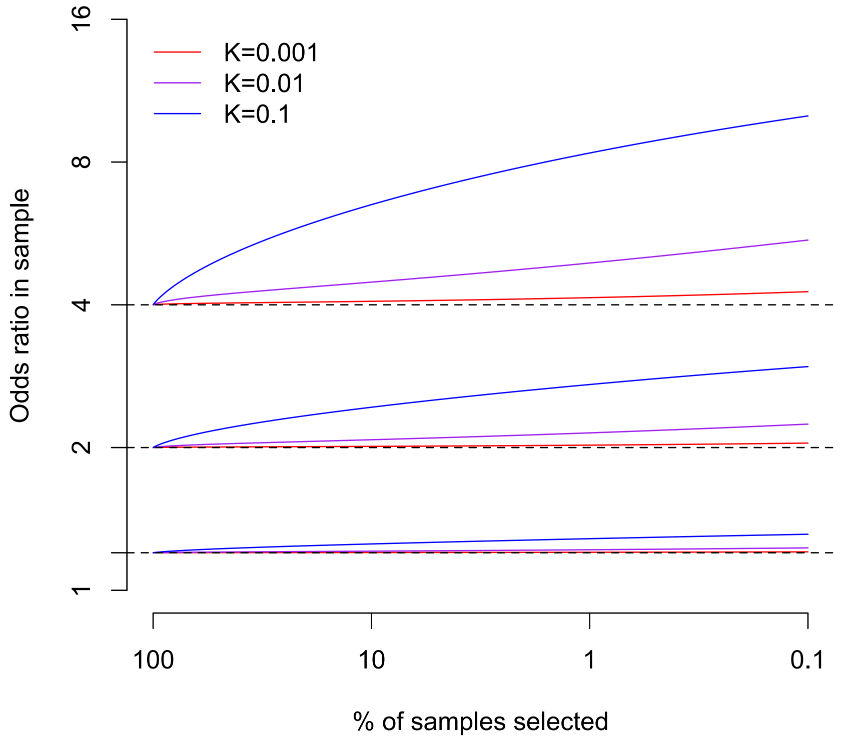

(a) Different prevalence

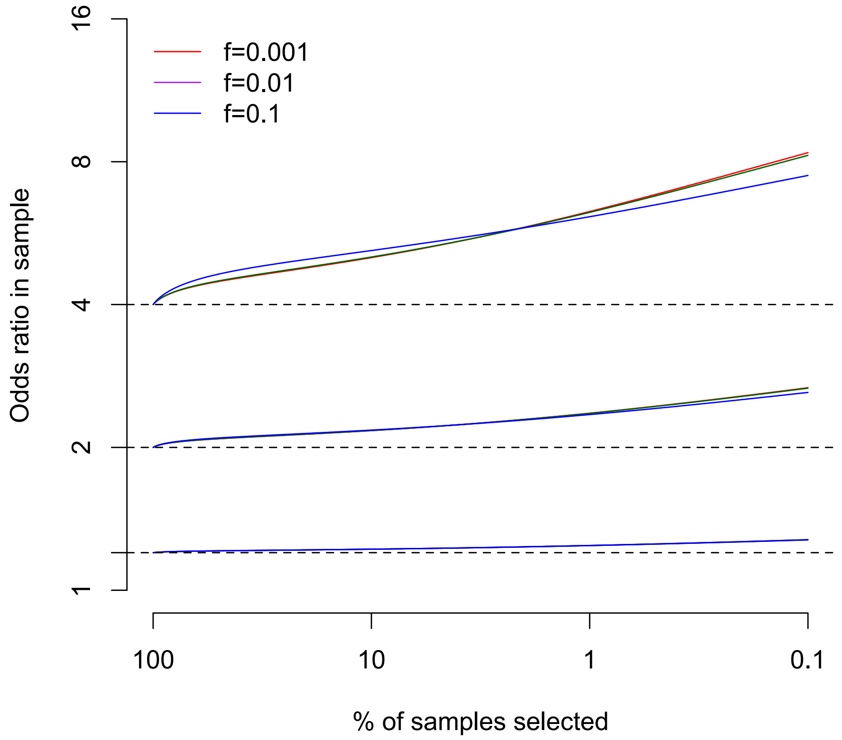

(b) Different risk allele frequency

Figure S2: Further plots on the relationship between the degree of selection based on risk score and the odds ratio, using different values of a) prevalence (K), with $h_p^2 = 0.1$ and $f = 0.01$ and b) risk allele frequency (f), with $h_p^2 = 0.25$ and $K = 0.01$.
